# Combined histone deacetylase inhibition and tamoxifen induces apoptosis in tamoxifen-resistant breast cancer models, by reversing Bcl-2 overexpression

**DOI:** 10.1186/s13058-015-0533-z

**Published:** 2015-02-25

**Authors:** Paromita Raha, Scott Thomas, K Ted Thurn, Jeenah Park, Pamela N Munster

**Affiliations:** Division of Hematology and Oncology, Department of Medicine, University of California, 1600 Divisadero Street, Room A722, Box 1770, San Francisco, CA 94115 USA

## Abstract

**Introduction:**

The emergence of hormone therapy resistance, despite continued expression of the estrogen receptor (ER), is a major challenge to curing breast cancer. Recent clinical studies suggest that epigenetic modulation by histone deacetylase (HDAC) inhibitors reverses hormone therapy resistance. However, little is known about epigenetic modulation of the ER during acquired hormone resistance. Our recent phase II study demonstrated that HDAC inhibitors re-sensitize hormone therapy-resistant tumors to the anti-estrogen tamoxifen. In this study, we sought to understand the mechanism behind the efficacy of this combination.

**Methods:**

We generated cell lines resistant to tamoxifen, named TAMR^M^ and TAMR^T^, by continuous exposure of ER-positive MCF7 and T47D cells, respectively to 4-hydroxy tamoxifen for over 12 months. HDAC inhibition, along with pharmacological and genetic manipulation of key survival pathways, including ER and Bcl-2, were used to characterize these resistant models.

**Results:**

The TAMR^M^ cells displayed decreased sensitivity to tamoxifen, fulvestrant and estrogen deprivation. Consistent with previous models, ER expression was retained and the gene harbored no mutations. Compared to parental MCF7 cells, ER expression in TAMR^M^ was elevated, while progesterone receptor (PGR) was lost. Sensitivity of ER to ligands was greatly reduced and classic ER response genes were suppressed. This model conveyed tamoxifen resistance through transcriptional upregulation of Bcl-2 and c-Myc, and downregulation of the cell cycle checkpoint protein p21, manifesting in accelerated growth and reduced cell death. Similar to TAMR^M^ cells, the TAMR^T^ cell line exhibited substantially decreased tamoxifen sensitivity, increased ER and Bcl-2 expression and significantly reduced PGR expression. Treatment with HDAC inhibitors reversed the altered transcriptional events and reestablished the sensitivity of the ER to tamoxifen resulting in substantial Bcl-2 downregulation, growth arrest and apoptosis. Selective inhibition of Bcl-2 mirrored these effects in presence of an HDAC inhibitor.

**Conclusions:**

Our model implicates elevated ER and Bcl-2 as key drivers of anti-estrogen resistance, which can be reversed by epigenetic modulation through HDAC inhibition.

**Electronic supplementary material:**

The online version of this article (doi:10.1186/s13058-015-0533-z) contains supplementary material, which is available to authorized users.

## Introduction

About 70% of all breast cancers express the estrogen receptor (ER). Commonly used therapies to treat these cancers either target the ER directly through selective ER modulators and downregulators (SERMs and SERDs); or diminish endogenous estrogen levels via ovarian ablation or the use of aromatase inhibitors. However, the emergence of hormone therapy resistance remains a significant hurdle, as almost 40% of women with metastatic, ER-positive disease progress despite the initial efficacy [1].

The evolution of hormone therapy resistance appears to involve multiple diverging mechanisms. Thus, understanding the complexity of resistance is crucial to identify novel targets and select biomarkers. Mechanisms associated with acquired resistance to hormone therapy include decrease or loss of ER expression or function; variation in ER-associated transcription factor recruitment; genetic mutations and epigenetic modulations; elevation and activation of the HER2 pathway; mutations and modulation of the PI3K/mTOR pathway; upregulation of cyclin D1 and loss of p16; or activation of Myc pathway [1-3].

Emerging data link epigenetic changes affecting ER expression and its target gene promoters, to acquired resistance [4,5]. Histone deacetylases (HDAC) and transferases (HAT) are chromatin modifiers that lead to epigenetic changes in the cell and have been implicated in the development of drug resistance in several cancers including breast. These enzymes regulate acetylation of histone and non-histone proteins, and thereby control key cellular processes including cell cycle progression, proliferation, survival, DNA repair and differentiation [6,7]. There have been several studies evaluating the role of HDAC inhibitors in both ER-positive and -negative settings [8,9]. However, in clinical studies, HDAC inhibitors have failed to show considerable anti-tumor activity as single agents in breast tumors [10]. As such, HDAC inhibitors have become an attractive constituent of combination regimens, including hormone therapy for the treatment of breast cancer [1].

Recently, we reported the first clinical study evaluating the co-administration of an HDAC inhibitor (vorinostat) with an anti-estrogen (tamoxifen) in advanced breast cancer patients. Clinical benefit was achieved in 40% of patients (19% objective response and 21% stable disease for more than 6 months) despite progression on multiple prior anti-estrogen therapies and chemotherapy [11]. Subsequently, the HDAC inhibitor, entinostat, was shown to reverse hormone therapy resistance when combined with the aromatase inhibitor exemestane [12]. Thus, HDAC inhibition appears to reestablish sensitivity to anti-estrogens in a subset of resistant tumors. However, the ability to identify these responding tumors is limited by the poor understanding of the mechanism that underlies its effectiveness.

In the current study, we thus sought to characterize the mechanism underpinning the effectiveness of inhibiting HDAC and ER activity in anti-estrogen-resistant breast cancer. We developed novel breast cancer cell lines that model acquired tamoxifen-resistant breast cancer (tamoxifen-resistant cells derived from MCF7 (TAMR^M^) and tamoxifen-resistant cells derived from T47D (TAMR^T^)). These models exhibit elevated ER, Bcl-2, and c-Myc expression and reduced p21 expression, which together result in enhanced cell proliferation and reduced susceptibility to cell death. Although ER is overexpressed, ligand-mediated ER transactivation is substantially reduced. HDAC inhibition is sufficient to reverse ER, c-Myc and p21 expression and inhibit proliferation. However, combined HDAC and ER inhibition is required for significant Bcl-2 downregulation and apoptotic induction. Thus, tumors that exhibit apoptotic resistance and impaired proliferation checkpoints may be candidates for combined HDAC and ER inhibition.

## Materials and methods

### Chemicals, antibodies and drugs

4-hydroxy tamoxifen (Tam) was purchased from Calbiochem (San Diego, CA, USA). Valproic acid and fulvestrant (Ful) were purchased from Sigma-Aldrich (St. Louis, MO, USA). Entinostat and ABT.263 were purchased from Selleck Chemicals LLC (Houston, TX, USA). PCI-24781 (PCI), vorinostat and panobinostat were gifts from Pharmacyclics Inc. (Sunnyvale, CA, USA), Aton Pharma Inc. and Novartis International (Basel, Switzerland), respectively. Antibodies against ER-α and p21 were purchased from Santa Cruz Biotechnology, Inc. (Santa Cruz, CA, USA). Antibodies against progesterone receptor (PGR), Bim, Bak, Bax, Bok, Bid, c-Myc and PARP were purchased from Cell Signaling Technology (Danvers, MA, USA). GAPDH and beta-actin antibodies were purchased from EMD Millipore (Billerica, MA, USA) and Abcam (Cambridge, MA, USA).

### Cell culture

MCF7 and T47D cells were purchased from American Type Culture Collection (Manassas, VA, USA) and maintained in Dulbecco’s modified Eagle’s medium (DMEM) (Thermo Fisher Scientific, Waltham, MA, USA) supplemented with 10% fetal bovine serum (Sigma-Aldrich), 2 mM glutamine, 50 unit/mL penicillin and 50 μg/ml streptomycin (Thermo Fisher Scientific). The above media was used for all experiments unless specified. The Tam-resistant cell lines TAMR^M^ and TAMR^T^ were generated by continuous exposure of MCF7 and T47D cells to increasing doses of Tam up to 10 μM and 6 μM, respectively, in complete DMEM and thereafter maintaining them in presence of 10 μM and 6 μM Tam. Cells were maintained at 37°C in a humidified atmosphere containing 5% CO_2_. Resistant cell lines were passaged for a maximum of 40 times, to minimize further evolution of the cell lines during this study.

### Colony-forming assay

Two hundred cells per well were seeded in a 12-well plate and treated with vehicle (DMSO), 200 nM PCI (P), 10 μM Tam (T) or the PCI-24781 and 4-hydroxy tamoxifen (PT) combination for 72 hours after which media with drugs were replaced with fresh media. Fourteen days later, cells were fixed with methanol, stained with 2% crystal violet and colonies were counted.

### Cell proliferation and viability assays

Proliferation assays were conducted using CellTiter 96 Cell Proliferation Assay (MTS) solution following the manufacturer’s protocol (Promega, Madison, WI, USA). BrdU incorporation assay was conducted using the assay kit from Cell Signaling Technology following the manufacturer’s recommendation. Viability was assayed by trypan blue dye exclusion, as previously described [13].

### Cell cycle analysis by flow cytometry

Cells were stained with propidium iodide (PI) using the Abcam kit (ab139418) according to the provided protocol. Briefly, cells were harvested, washed in ice-cold phosphate-buffered saline (PBS) and fixed in 70% ethanol for 30 minutes at 4°C. After two PBS washes, cells were treated with RNase A for 15 minutes at 37°C, stained with 5 μg/mL PI in PBS and assayed with a FACS Calibur (BD Biosciences, San Jose, CA, USA) flow cytometer using Cell Quest software. The cell cycle distribution was analyzed using BD CellQuest™ Pro Analysis software (BD Biosciences).

### Luciferase reporter gene assay

Cells were trypsinized and collected in phenol red-free media supplemented with 5% charcoal dextran stripped serum (CDSS) (Invitrogen, Carlsbad, CA, USA) and transfected with the ERE-tk-109-luciferase plasmid using Lipofectamine™ LTX (Invitrogen). Transfected cells were incubated in 96-well plates for 5 hours, after which ligands prepared in serum-free media were added to the cells and incubated for 20 hours. Media was then discarded, and 50 μl of Promega bright-glo luciferase substrate dissolved in lysis buffer was added to the cells before recording luminescence using a Veritas Microplate Luminometer (Promega).

### Western immunoblotting

Proteins were extracted in lysis buffer (0.1% SDS, 1% Triton X-100, 50 mM Tris-HCl pH 7.4, 150 mM NaCl, 10% glycerol, 1X Halt protease and phosphatase inhibitor cocktail (Thermo Fisher Scientific)), separated on 4 to 12% Bis-Tris Nu-PAGE gels and transferred to Immobilon-P polyvinylidene fluoride (PVDF) microporous membrane (EMD Millipore) at 100 V for 1 hour. Membranes were blocked using 5% (w/v) non-fat dry milk in Tris-buffered saline containing 0.1% Tween 20 (TBST) and subsequently probed with primary antibodies followed by horseradish peroxidase-linked secondary antibodies and visualized using the ECL Plus Western Blotting Detection System (GE Healthcare, Little Chalfont, UK).

### siRNA depletion

Small interfering RNA (siRNA) duplexes for ESR1 (ID# 42835) and BCL-2 (ID# 214532) mRNA depletion were purchased from Applied Biosystems (Carlsbad, CA, USA). Cells were transfected with siRNA duplexes by nucleofection using the Nucleofector Transfection Kit according to the manufacturer’s protocol (Amaxa, Gaithersburg, MD, USA). The silencer negative control #2 from Applied Biosystems was used as a transfection control and referred to as scramble (Sc).

### mRNA expression analysis

Total RNA was purified from cells using the Qiagen RNeasy Kit (Valencia, CA, USA). cDNA was generated using the iScript cDNA synthesis kit (Bio-Rad Labs Inc., Hercules, CA, USA). Taqman expression assays for ESR1 (ID# Hs00174860_m1), PGR (Hs01556702_m1), TFF1 (ID# Hs00907239_m1), BCL-2 (ID# Hs00608023_m1), CDH1 (ID# Hs01023894_m1), GREB1 (ID# Hs00536409_m1), H.CTSD (ID# Hs00157205_m1), TRIM25 (ID# Hs01116121_m1), and c-MYC (ID# Hs00153408_m1) were purchased from Applied Biosystems. Expression was determined using an ABI 7900 HT Thermocycler (Applied Biosystems) and normalized to b-glucuronidase (h.Gus).

### *In vivo* studies

Animal studies were conducted according to a UCSF Laboratory Animal Resource Center (LARC) protocol (AN090303). This protocol was approved by the UCSF Institutional Animal Care and Use Committee (IACUC) accredited by Association for Assessment and Accreditation of Laboratory Animal Care International (#001084). Four- to six-week-old female nude athymic Crl;NU(NCr)-Foxn1^nu^ mice (Charles River Laboratories, Wilmington, MA, USA) were used for the study. MCF7 and TAMR^M^ cells were implanted subcutaneously following subcutaneous implantation of a 60-day release estradiol (E2) pellet. For each cell line, two cohorts of five mice received subcutaneous administrations, 5/7 days, with either 50 μL of 10 mg/mL tamoxifen citrate in peanut oil or vehicle. At the conclusion of the study, tumors were harvested. Protein was extracted from tumors using a mortar and pestle in the presence of liquid nitrogen. Ground tumors were incubated with cell lysis buffer (0.1% SDS, 1% Triton X-100, 50 mM Tris-HCl pH 7.4, 150 mM NaCl, 10% glycerol, 1X Halt protease and phosphatase inhibitor cocktail), syringe passaged, and lysate cleared by centrifugation.

### Statistical analysis

Data are expressed as averages, with the standard error of mean (±S.E.M.) indicated. A two-sided non-paired Student’s *t* test was used to determine differences between two groups with *P* <0.05 considered statistically significant.

## Results

### Tamoxifen-resistant cells display accelerated growth rate and decreased sensitivity to fulvestrant

**Figure 1:**
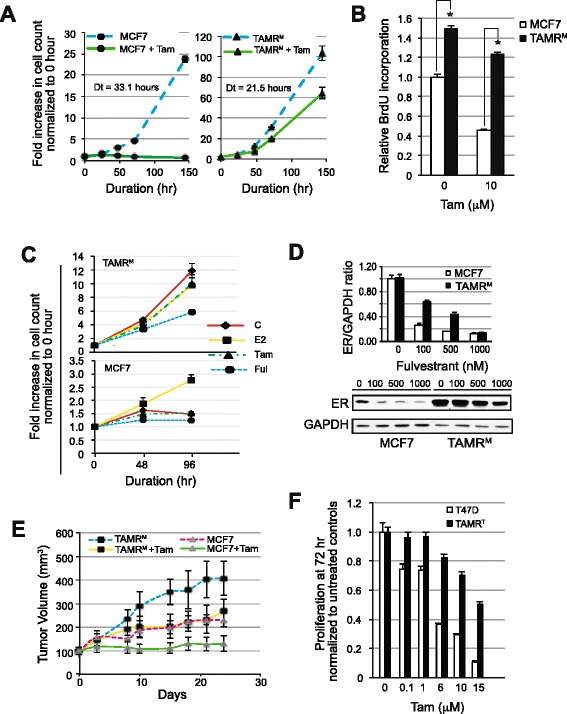
**Tamoxifen-resistant cells display accelerated growth rate and decreased sensitivity to fulvestrant. (A)** Cells were grown in complete media in the presence or absence of 10 μM 4-hydroxy tamoxifen (Tam) for 24, 48, 72 and 144 hours, and **(C)** in estradiol (E2)-free media for 48 and 96 hours and supplemented with vehicle (C) (DMSO), 1 nM E2, 1 μM Tam and 0.1 μM fulvestrant (Ful). Fold increase in cell counts normalized to zero hour counts of respective cell lines are represented. **(B)** Cells were grown in the presence of DMSO or 10 μM Tam in complete media. BrdU substrate was added 48 hours after drug treatment and assayed after 24 hours. **(D)** Cells were treated with increasing Ful concentrations for 24 hours and evaluated for estrogen receptor (ER) protein. Presented is a representative western blot. The bar graph represents densitometry analysis of the ER/GAPDH ratio, normalized to the respective untreated controls, from two independent experiments. **(E)** Tumor volumes of MCF7 and tamoxifen-resistant cells derived from MCF7 (TAMR^M^) cell mouse xenografts treated over time with vehicle or Tam are represented. **(F)** T47D and tamoxifen-resistant cells derived from T47D (TAMR^T^) cells were treated with increasing Tam doses for 72 hours. Proliferation was determined and normalized to respective untreated controls. For graphs, averages are presented with error bars indicating the standard error of the mean. Asterisk (*) indicates significant difference (*P* value < 0.05), compared to control group. Dt, doubling time.

Previous studies have shown that continuous exposure of ER-positive breast cancer cells to Tam results in suppression of classical ER genomic signaling activity without loss of ER expression [14-17]. This suggests a direct role for the ER in driving resistance [18]. To mimic the emergence of hormone therapy resistance, we generated *in vitro* Tam-resistant cell lines TAMR^M^ and TAMR^T^ (see Materials and methods). Resistance to Tam was reflected in TAMR^M^ and MCF7 total cell counts, recorded over time with and without 10 μM Tam treatment and expressed as fold increase over time compared to baseline (0 h) (Figure 1A). In the absence of Tam, TAMR^M^ cells grew considerably faster than parental MCF7 cells, as illustrated by the doubling times (Dts, 21.5 versus 33.1 hours). Treatment with 10 μM Tam arrested MCF7 cell growth. However, with the same concentration of Tam, TAMR^M^ cell growth was only modestly reduced (23.9% reduction in doubling time). Consistent with elevated proliferation rate, TAMR^M^ cells exhibited greater BrdU incorporation compared to MCF7 cells, both in the absence and presence of Tam (Figure 1B). To test whether TAMR^M^ cells had altered sensitivity to estrogen and the anti-estrogen fulvestrant, cells were incubated for 48 hours in estrogen-free media and then treated with vehicle (C), E2 1 nM, Tam 1 μM or Ful 0.1 μM. As expected, E2 stimulated the growth of MCF7 cells, whereas the absence of E2 or the presence of anti-estrogens Tam or Ful inhibited growth (Figure 1C). In contrast, the presence or absence of E2 or Tam did not significantly affect TAMR^M^ cell growth. Furthermore, treatment with Ful only partially inhibited TAMR^M^ cell growth. Thus, consistent with previous reports, development of Tam resistance in TAMR^M^ cells resulted only in partial cross-resistance to Ful [19,20]. Treatment with increasing Ful concentrations for 24 hours depleted ER protein in both cell lines (Figure 1D). However, comparable doses of Ful up to 500 nM resulted in substantially more ER loss in MCF7 compared to TAMR^M^ cells, consistent with the growth assay. This suggested the TAMR^M^ cells were less sensitive to Ful inhibition compared to MCF7 cells. The increased proliferation and decreased sensitivity of TAMR^M^ cells to Tam was also observed in *in vivo* tumor xenografts (Figure 1E). Similar to cells grown *in vitro*, TAMR^M^-derived tumors did not require E2 for growth, unlike MCF7 cells (Figure S1 in Additional file 1). The relative proliferation of TAMR^T^ cells as compared to T47D cells, treated with increasing Tam doses in complete media for 72 hours demonstrates the Tam resistance of this TAMR^T^ cell line (Figure 1F).

### Tamoxifen-resistant cells display altered gene expression profile

**Figure 2:**
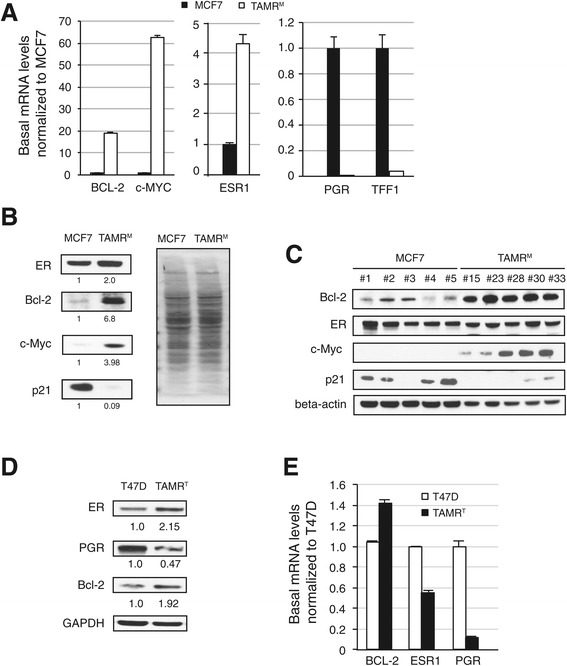
**Tamoxifen-resistant cells display altered gene expression profile. (A)** MCF7 and tamoxifen-resistant cells derived from MCF7 (TAMR^M^) basal mRNA levels of ESR1, BCL-2, c-MYC, PGR and TFF1 are represented normalized to h.glucoronidase. **(B)** Comparison of estrogen receptor (ER), Bcl-2, c-Myc and p21 baseline expression in MCF7 and TAMR^M^ by western blot. As a suitable loading control protein could not be identified, the Ponceau S stained membrane is presented to demonstrate equal protein loading. Bands were quantified by densitometry. The value below each band represents quantified protein in respective cell line normalized to MCF7. **(C)** Western blot analysis of ER, Bcl-2, c-Myc and p21 expression levels in untreated MCF7 and TAMR^M^ tumor xenografts are presented. Comparison of T47D and tamoxifen-resistant cells derived from T47D (TAMR^T^) basal levels of ER, progesterone receptor (PGR) and Bcl-2 protein **(D)** and mRNA **(E)** are presented where protein expression is normalized to GAPDH, while mRNA expression is normalized to h. glucoronidase. ImageJ software was used for densitometry analysis of western blots. For graphs, averages are presented with error bars indicating the standard error of the mean.

Several mechanisms of resistance have been attributed to the development of acquired hormone resistance, including altered expression of key proteins regulating proliferation and death. Consistent with prior preclinical models and in the clinic, ER expression was maintained in both our tamoxifen-resistant cell lines [21,22]. Evaluation of ER expression in TAMR^M^ cells revealed that both mRNA (ESR1) and protein were higher compared to MCF7 cells, 4- and 2-fold, respectively (Figure 2A and B).

To determine whether increased ER expression affected its activity, ER target gene expression was examined. Despite the elevated levels of ER in both resistant models, expression of classic ER response genes (for example PGR and TFF1) were substantially reduced (Figure 2A and E). It has been previously shown that the pro-growth and survival signals mediated by estrogen signaling are in part through the regulation of c-Myc, p21 and Bcl-2 expression [23-25]. Consistent with the higher proliferation capacity of TAMR^M^ cells, the major cell cycle checkpoint protein p21 was suppressed (approximately 90%) (Figure 2B) and c-Myc substantially elevated (approximately 300%) compared to MCF7 cells. Furthermore, Bcl-2 expression increased nearly 600%. Consistent with these *in vitro* findings, the TAMR^M^ xenografts expressed higher Bcl-2 and c-Myc proteins and lower p21 protein compared to the MCF7 xenografts. However there was no significant increase in ER protein in the TAMR^M^ xenografts (Figure 2C). Comparing the basal level of proteins in the TAMR^T^ cells, we found that these resistant cells also displayed higher ER and Bcl-2 proteins and substantially reduced PGR protein as compared to T47D parental cells (Figure 2D). Basal mRNA levels of BCL-2 and PGR corresponded with the proteins, but ESR1 mRNA was not elevated in the TAMR^T^-resistant cells although ER protein was found to be elevated (Figure 2E). This suggests that the relative mechanisms leading to ER protein upregulation likely differ in the two resistant models.

### ER-mediated transactivation and ligand sensitivity is altered in TAMR^M^ cells

**Figure 3:**
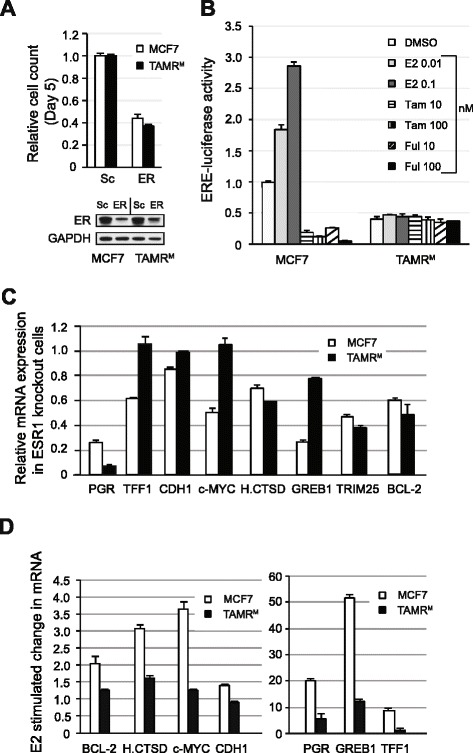
**ER-mediated transactivation and ligand sensitivity is altered in TAMR** ^**M**^ **cells. (A)** MCF7 and tamoxifen-resistant cells derived from MCF7 (TAMR^M^) cells were transfected with ESR1-directed small interfering RNA (siRNA) or scramble and total cell count was monitored after 5 days. Western blot of ER knockdown is presented. **(B)** MCF7 and TAMR^M^ cells were transfected with an ERE-tk-luc plasmid and treated with either vehicle (DMSO); 0.01 or 0.1 nM estradiol (E2); 10 or 100 nM 4-hydroxy tamoxifen (Tam); 10 or 100 nM fulvestrant (Ful) for 20 hours and assayed for luciferase activity **(C)** MCF7 and TAMR^M^ cells were transfected with ESR1-directed siRNA and assayed for mRNA expression of estrogen receptor (ER) target genes. mRNA expression is normalized to scramble control for each cell line. **(D)** MCF7 and TAMR^M^ cells were incubated in phenol red-free media supplemented with 5% charcoal dextran stripped serum (CDSS) for 72 hours after which 0 nM or 0.1 nM E2 was added for 24 hours and mRNA expression of depicted genes was quantified and represented as fold change upon E2 stimulation versus no E2. For graphs, averages are presented with error bars indicating the standard error of the mean.

External stimulation by E2 results in ER dimerization and activation of estrogen response elements (EREs) or co-factor recruitment to non-ERE sites, which drives ER target gene expression and proliferation of ER-positive MCF7 cells. To determine the role of the ER in the accelerated growth rate of TAMR^M^ cells, we conducted siRNA-mediated knockdown of ER (ESR1) mRNA in both MCF7 and TAMR^M^ cells, and found comparable ER depletion resulted in an approximately 60% growth reduction in both cell lines (Figure 3A). This suggests the ER remains an important mediator of cell growth in TAMR^M^ cells despite substantial reduction in sensitivity to estrogen and anti-estrogens (Figure 1). We next tested whether this reduced sensitivity was the result of altered ER-mediated signaling. To study relative ligand sensitivity of the ER in TAMR^M^ and MCF7 cells, we transfected each with a luciferase reporter plasmid (pERE-luc-tk) driven by a promoter containing an ERE. As expected, treatment of MCF7 cells with E2 increased luciferase activity with increasing dose, while Tam and Ful decreased luciferase activity below basal levels. On the other hand, luciferase activity in TAMR^M^ cells remained unaffected by E2 or anti-estrogen treatment (Figure 3B). To evaluate whether the ER-mediated target gene transcription was altered in TAMR^M^ cells, mRNA from a set of known ER-responsive genes (for example PGR, TFF1, CDH1, c-MYC, H.CTSD, GREB1, TRIM25, and BCL2), were quantified following siRNA-mediated depletion of the ER and compared to similar depletion in MCF7 cells (Figure 3C). In MCF7 cells, ER depletion resulted in downregulation of all target mRNAs to varying degrees. Interestingly in TAMR^M^ cells, all but three transcripts (for example TFF1, CDH1 and c-MYC) were downregulated following ER depletion, suggesting that, independent of E2, ER continued to regulate gene expression in TAMR^M^ cells. To test whether ER-mediated expression was truly E2 independent, TAMR^M^ cells were stimulated with E2 and mRNA levels of these select genes were quantified and compared to MCF7 cells (Figure 3D). Consistent with siRNA-mediated depletion of ER, E2 stimulation did not significantly increase TFF1 and CDH1 mRNA, while c-MYC, H.CTSD and BCL-2 mRNA were only slightly increased. PGR and GREB1 mRNA were significantly increased with E2 stimulation; however, their increase was substantially less than the increase observed in MCF7 cells. Taken together, these results suggest that ER remains important for TAMR^M^ cell growth, but has differential transactivation properties at target genes compared to MCF7 cells. Furthermore, the ER in TAMR^M^ cells has significantly reduced sensitivity to E2.

### Combined HDAC and ER inhibition reduces proliferation and viability of tamoxifen-resistant cells

**Figure 4:**
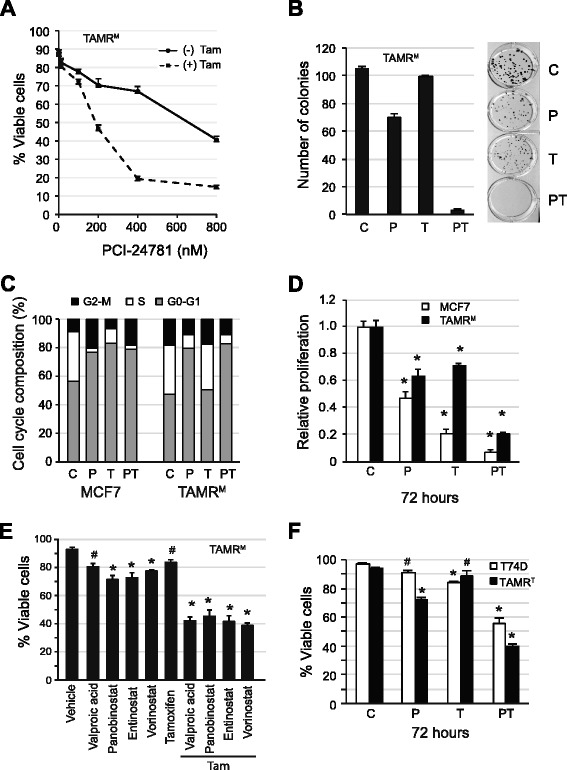
**Combined HDAC and ER inhibition reduces proliferation and viability of tamoxifen-resistant cells. (A)** Tamoxifen-resistant cells derived from MCF7 (TAMR^M^) cells were treated with and without 10 μM 4-hydroxy tamoxifen (Tam) and increasing concentrations of PCI-24781(PCI) for 72 hours and assayed for viability using a trypan blue dye exclusion assay. **(B)** TAMR^M^ cells were treated with either vehicle (C), 200 nM PCI (P), 10 μM Tam (T) or the combination of PCI-24781 and 4-hydroxy tamoxifen (PT) for 72 hours and evaluated by colony formation assay. Colonies were counted after 2 weeks and considered formed if they contained approximately >100 cells. MCF7 and TAMR^M^ cells were treated with vehicle (C), 200 nM PCI (P), 10 μM Tam (T) or the combination (PT) for **(C)** 24 hours and evaluated for cell cycle composition by flow cytometry or **(D)** 72 hours and evaluated for relative proliferation by MTS assay. **(E)** TAMR^M^ cells were treated with either 0.5 mM valproic acid, 25 nM panobinostat, 1 μM vorinostat or 1 μM entinostat in presence and absence of 10 μM Tam for 72 hours and assayed for viable cells. **(F)** T47D and tamoxifen-resistant cells derived from T47D (TAMR^T^) cells were treated as in **(D)** and assayed for viable cells. Asterisk (*) indicates significant difference (*P* value <0.05) whereas (^#^) indicates insignificant difference (*P* value >0.05), compared to control group. HDAC, histone deacetylase.

We have previously shown that the addition of an HDAC inhibitor to hormonal therapy can induce apoptosis in breast cancer cells and reverse hormone therapy resistance in clinical studies [11,26,27]. For this study, the potent hydroxamic-type pan-HDAC inhibitor PCI-24781 (abexinostat) was primarily used. To evaluate the efficacy of this combination, TAMR^M^ cells were treated with increasing concentrations of PCI, with and without 10 μM Tam, and assayed for viability after 72 hours (Figure 4A). At clinically feasible concentration of PCI (200 nM), combination with Tam resulted in cell death in about 54% of the TAMR^M^ cells, greater than the additive effect of either agent alone. The PT drug combination was most effective in inhibiting colony formation. (Figure 4B). Consistent with an elevated proliferation rate (Figure 1A), untreated TAMR^M^ cells formed colonies within 14 days, while MCF7 cells failed to form detectable colonies within this time (DNS).

In MCF7 cells, combined HDAC and ER inhibition results in reduced proliferation, with cells accumulating in G1 of the cell cycle. As such, the effect of PCI and Tam alone and in combination on cell cycle progression after 24-hour treatment was examined. Untreated TAMR^M^ cells had a 2-fold greater G2-M population (Figure 4C) compared to parental MCF7 cells, consistent with accelerated growth. Unlike in MCF7 cells, Tam treatment did not result in a G1 shift in the TAMR^M^ cells. Treatment with PCI, however, resulted in a similar G1 accumulation for both TAMR^M^ and MCF7 cells. When treated in combination, both MCF7 and TAMR^M^ cells exhibited cell cycle distributions comparable to PCI treatment alone. We next evaluated the effect of the PT combination on cell proliferation after 72 hours of treatment. PCI alone comparably restricted growth of both cell lines. While Tam alone significantly reduced the growth of parental cells (80%), it only modestly reduced TAMR^M^ cell growth (30%) (Figure 4D). The combination treatment resulted in an enhanced anti-proliferative effect, comparable in both cell lines. The combined effect of HDAC and ER inhibition on TAMR^M^ cell viability was not limited to PCI. Combinations with Tam and several other HDAC inhibitors, including valproic acid, panobinostat, entinostat and vorinostat, resulted in a greater than additive reduction in cell viability (Figure 4E). To exclude the possibility of any non-ER mediated effect of Tam we have demonstrated enhanced apoptotic cell death by HDAC inhibition in ER-depleted TAMR^M^ cells (Figure S2 in Additional file 2). We further evaluated whether treatment with PCI and Tam could induce cell death in TAMR^T^ cells. After 72 hours of treatment, PCI alone induced 30% cell death, Tam alone did not lead to significant death, and the combination resulted in 60% cell death, suggesting an enhanced effect in presence of both drugs (Figure 4F). In summary, combined HDAC and ER inhibition induced growth arrest and cell death in our acquired Tam-resistant breast cancer models. While growth arrest appeared to be primarily the effect of HDAC inhibition, significant cell death required both agents.

### Combined HDAC and ER inhibition reverses altered Bcl-2, c-Myc and p21 expression

**Figure 5:**
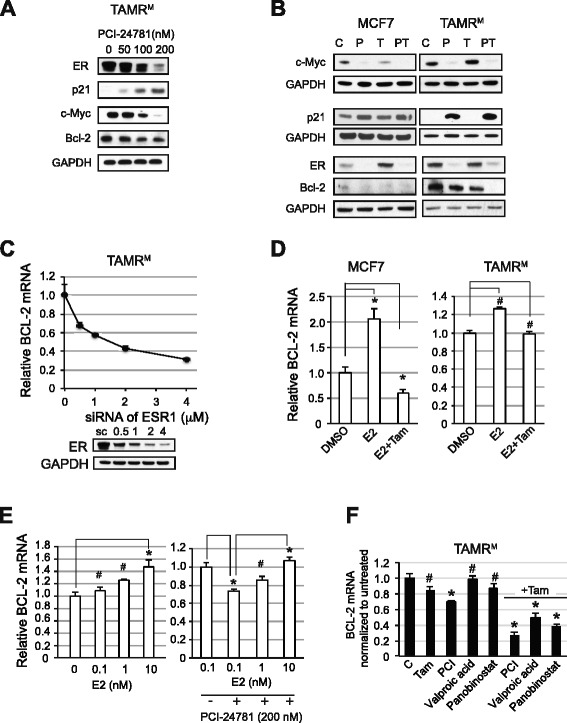
**Combined HDAC and ER inhibition reverses altered Bcl-2, c-Myc, and p21 expression. (A)** Tamoxifen-resistant cells derived from MCF7 (TAMR^M^) cells were treated with increasing concentrations of PCI-24781 (PCI) for 24 hours and evaluated for estrogen receptor (ER), p21, c-Myc, Bcl-2 and GAPDH expression by western blot. **(B)** MCF7 and TAMR^M^ cells were treated with vehicle (C), 200 nM PCI (P), 10 μM 4-hydroxy tamoxifen (Tam) (T), or the combination of PCI-24781 and 4-hydroxy tamoxifen (PT) for 48 hours and evaluated for ER, c-Myc, p21 and Bcl-2 expression by Western blot. **(C)** TAMR^M^ cells were transfected with increasing concentrations of small interfering RNA (siRNA) for ESR1 and 24 hours later analyzed for BCL-2 expression. The blot depicts loss of ER protein with increasing concentrations of siRNA directed to ER. MCF7 and TAMR^M^ cells were incubated in estrogen-free media for 48 hours and **(D)** treated with vehicle (DMSO) (C), 0.1 nM estradiol (E2) or 0.1 nM E2 + 10 μM Tam for 24 hours and **(E)** TAMR^M^ cells were treated with increasing doses of E2 (0.1, 1 or 10 nM) in presence or absence of 200 nM PCI for 24 hours and relative BCL-2 mRNA expression was measured. **(F)** Relative BCL-2 mRNA expression was measured in complete media 24 hours post treatment with vehicle (C), 10 μM Tam, 200 nM PCI, 2 mM valproic acid or 20 nM panobinostat, in absence and presence of Tam. Asterisk (*) indicates significant difference (*P* value <0.05) whereas (^#^) indicates insignificant difference (*P* value >0.05), compared to control group. HDAC, histone deacetylase.

As shown in Figure 2, the major anti-apoptotic protein, Bcl-2, and proliferation driver, c-Myc, are significantly overexpressed in TAMR^M^ cells. Additionally, a key cell cycle break, p21, is downregulated. We thus hypothesized that the efficacy of combined HDAC and ER inhibition may be the result of reversing the altered expression of these key proteins. To test this hypothesis, we first treated TAMR^M^ cells with increasing concentrations of PCI for 24 hours and evaluated protein expression of ER, p21, c-Myc, and Bcl-2 (Figure 5A). With increasing dose, ER and c-Myc protein were significantly downregulated, while p21 was upregulated. Although modest, Bcl-2 protein was reproducibly reduced when treated with 100 nM or more PCI. As HDACs regulate both ER protein stability and transcription, the effect of PCI treatment on ER mRNA was evaluated. As seen previously with MCF7 cells, HDAC inhibition using PCI reduced TAMR^M^ cell transcription of ER mRNA in a dose- and time-dependent manner (Figure S3 in Additional file 3). To determine the effect of combined HDAC and ER inhibition on the expression of these key proteins, MCF7 and TAMR^M^ cells were treated with vehicle, 200 nM PCI, 10 μM Tam, or the combination for 48 hours and evaluated for c-Myc, p21, and Bcl-2 expression (Figure 5B). In MCF7 cells, treatment with either single agent reduced c-Myc and Bcl-2 and increased p21 expression. In TAMR^M^ cells, PCI elicited similar changes to the expression of these proteins compared to MCF7 cells, while Tam had no effect on p21 or c-Myc expression, but slightly reduced Bcl-2. PCI treatment reduced ER in both cell lines. Though Tam treatment stabilized ER in MCF7 cells, ER was unaltered in TAMR^M^ cells. With the PT combination, p21 expression was further increased in MCF7 cells compared to single agent treatment. Due to the significant single agent effect on c-Myc and Bcl-2 expression, a combination effect was difficult to determine in MCF7 cells. In TAMR^M^ cells, combination treatment resulted in similar changes to ER, p21 and c-Myc expression compared to PCI treatment alone. In contrast, PT combination resulted in a greater than additive decrease in Bcl-2 expression.

ER regulates BCL-2 transcription in TAMR^M^ cells (Figure 3C) similar to that observed in parental MCF7 cells [23]. Depletion of ER using increasing concentrations of ESR1-directed siRNA (Figure 5C) resulted in a commensurate decrease of BCL-2 mRNA in the resistant cells, demonstrating that the ER continues to regulate BCL-2 transcription. However, physiological levels of E2 (0.1 nM) or E2 + Tam (0.1 nM E2 and 10 μM Tam) fail to modulate BCL-2 transcription in TAMR^M^ cells, in contrast to MCF7 cells (Figure 5D). To find out whether this was due to reduced sensitivity of the ER to ligands, TAMR^M^ cells were treated with increasing concentrations (0.1, 1 and 10 nM) of E2. A statistically significant increase in BCL-2 mRNA was observed with only 10 nM E2, but not with lower E2 doses (Figure 5E). To examine the influence of PCI on the ER response to E2, BCL-2 mRNA was evaluated in the presence of 200 nM PCI and increasing E2 doses. PCI (200 nM) slightly reduced BCL-2 mRNA, potentially by downregulating ER expression, which was significantly reversed with the addition of a very high E2 (10 nM) dose (Figure 5E). This suggested that the ER in TAMR^M^ cells are less sensitive to E2 and require more E2 to modulate expression of BCL-2. To evaluate whether HDAC inhibition enabled Tam to reduce ER-mediated BCL-2 transcription, TAMR^M^ cells were treated with DMSO (C), 10 μM Tam, 200 nM PCI or PT for 24 hours and BCL-2 mRNA expression was determined (Figure 5F). Tam alone had no significant effect on BCL-2 mRNA. Treatment with PCI reduced BCL-2 mRNA modestly (approximately 30%), even though the ER was reduced by approximately 75% at this dose. Higher concentrations of PCI, beyond the clinically feasible dose of 200 nM, did significantly deplete BCL-2 mRNA (Figure S4 in Additional file 4), consistent with the increased rates of cell death at those doses. However, combined PT treatment reduced BCL-2 mRNA levels by approximately 70% (Figure 5F). To determine whether these effects were specific to PCI, other HDAC inhibitors, valproic acid (2 mM) and panobinostat (20 nM) were evaluated. Both inhibitors alone did not significantly decrease BCL-2 mRNA, however, in combination with Tam, BCL-2 mRNA was significantly reduced.

### PCI and Tam combination alters expression of pro-apoptotic proteins and promotes apoptosis

**Figure 6:**
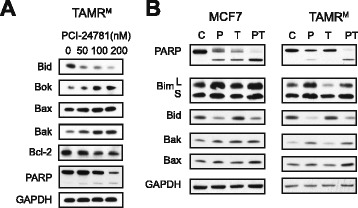
**PCI and Tam combination alters expression of pro-apoptotic proteins and promotes apoptosis. (A)** Tamoxifen-resistant cells derived from MCF7 (TAMR^M^) cells were treated with increasing doses of PCI-24781 (PCI) for 72 hours and expression of indicated proteins were evaluated by western blot. **(B)** MCF7 and TAMR^M^ cells were treated with vehicle (C), 200 nM PCI (P), 10 μM 4-hydroxy tamoxifen (Tam) (T) or the combination of PCI-24781 and 4-hydroxy tamoxifen (PT) for 72 hours and PARP cleavage and expression of pro-apoptotic proteins was evaluated by western blot.

The combination of HDAC and ER inhibition causes cell death in MCF7 and TAMR^M^ cells (Figure 4). HDAC inhibitors are known to induce pro-apoptotic proteins, which we have previously shown with PCI-treated MCF7 cells [13]. Thus, we hypothesized that downregulation of Bcl-2 (Figure 5) combined with upregulation of pro-apoptotic factors together drive TAMR^M^ cells into apoptosis. To test this hypothesis, TAMR^M^ cells were treated with increasing concentrations of PCI for 72 hours and evaluated for expression changes in key apoptotic proteins (Figure 6A). In response to increasing PCI doses, several pro-apoptotic proteins, including Bax, Bak, Bok and cleavage of Bid, exhibited pro-apoptotic expression changes accompanied by a modest increase in PARP cleavage. To evaluate the effect of combined HDAC and ER inhibition on the expression of these proteins and the induction of apoptosis, MCF7 and TAMR^M^ cells were treated with 200 nM PCI (P), 10 μM Tam (T), or the combination for 72 hours. Changes to apoptotic proteins in both MCF7 and TAMR^M^ cells were primarily seen with PCI treatment and not Tam, with the combination treatment eliciting similar changes to PCI treatment alone (Figure 6B). An exception seen in both MCF7 and TAMR^M^ cells was Bax expression, which exhibited a greater than additive increase. Furthermore PARP cleavage was significantly enhanced in both cell lines with PT combination, confirming apoptotic cell death. Taken together these results suggest that HDAC inhibition can reverse Tam resistance by depletion of the major pro-survival protein Bcl-2 and upregulation of pro-apoptotic factors, driving cells into apoptosis.

### Causal interaction of HDAC and BCL-2 in survival of TAMR^M^ cells

**Figure 7:**
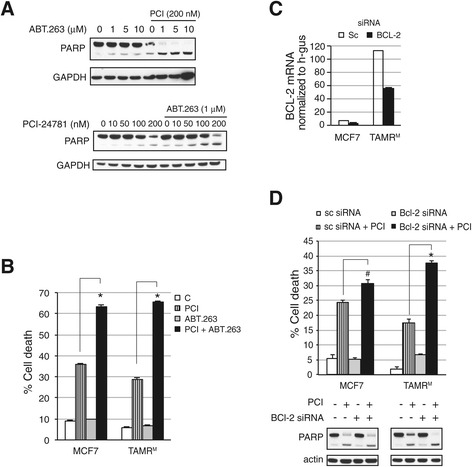
**Causal interaction of HDAC and BCL-2 in survival of TAMR** ^**M**^ **cells. (A)** Tamoxifen-resistant cells derived from MCF7 (TAMR^M^) cells were treated with increasing doses of ABT.263 in presence of 200 nM PCI-24781 (PCI) or increasing doses of PCI in presence of 1 μM ABT.263 and assayed by western blot for PARP cleavage. **(B)** MCF7 and TAMR^M^ cells were treated with vehicle (C), 200 nM PCI, 1 μM ABT.263 or the combination for 96 hours and analyzed for cell death. **(C)** MCF7 and TAMR^M^ cells were transfected with either scramble or a Bcl-2-specific small interfering RNA (siRNA) and extent of knockdown is represented by BCL-2 mRNA levels. **(D)** Treatment of the transient BCL-2 knockdown MCF7 and TAMR^M^ cells were treated with 200 nM PCI for 72 hours and analyzed for cell death and PARP cleavage. Asterisk (*) indicates significant difference (*P* value <0.05) whereas (^#^) indicates insignificant difference (*P* value >0.05) compared to control group. HDAC, histone deacetylase.

Several studies have demonstrated that overexpression of the pro-survival protein Bcl-2 confers resistance to apoptosis by apoptotic inducers in several cancers, including breast [28-30]. Similarly, apoptosis induction in TAMR^M^ was much lower compared to MCF7 cells when challenged with increasing doses of the apoptotic stress inducers, Tam or the anthracycline doxorubicin (Figure S5 in Additional file 5). To establish Bcl-2 as a major contributor to apoptotic resistance in TAMR^M^ cells, the Bcl-2 family member inhibitor ABT.263 was employed. Treatment of TAMR^M^ cells with doses of ABT.263 up to 10 μM resulted in modest PARP cleavage (Figure 7A), consistent with previous reports that demonstrate similar inhibitors alone are insufficient to induce apoptosis in breast cancer cells [31]. However, when combined with PCI (200 nM), 1 μM ABT.263 was sufficient to substantially induce PARP cleavage and cell death in TAMR^M^ cells (Figure 7A and B). Furthermore, combined ABT.263 and PCI treatment resulted in cell death comparable to combined Tam and PCI treatment in both MCF7 and TAMR^M^ cells (Figure 7B). To specifically implicate Bcl-2 as the key target, Bcl-2 was first depleted in TAMR^M^ cells. Analysis of mRNA expression revealed about 50% transient knockdown of BCL-2 mRNA (Figure 7C). These cells were then treated with PCI for 72 hours. Combined Bcl-2 depletion and HDAC inhibition was sufficient to induce significant cell death in TAMR^M^ cells (Figure 7D). However, genetically depleting BCL-2 in MCF7 cells followed by PCI treatment did not lead to additional cell death compared to PCI treatment alone. Together, these results demonstrate that Bcl-2 and HDAC inhibition is sufficient to induce cell death in TAMR^M^ cells.

## Discussion

The emergence of Tam resistance is almost inevitable in breast cancer. To aid in the development of novel therapeutics for this space, we generated breast cancer cell line models with substantially decreased sensitivity to Tam. Here we report Tam-resistant cell lines that maintain ER expression, but lose PGR expression. This receptor status is often seen in breast tumors and is associated with a relatively poor outcome. Our Tam-resistant breast cancer models exhibit ligand-independent growth and altered ER-mediated genomic signaling. We demonstrate that the ER is directly involved in controlling the pro-survival protein Bcl-2 by modulating its transcript. Our data further show that the addition of an HDAC inhibitor to the anti-estrogen Tam leads to inhibition of proliferation, prevention of colony formation, and induction of apoptotic cell death by downregulating Bcl-2 and upregulating pro-apoptotic proteins. HDAC inhibition in the presence of Tam results in depletion of ER and Bcl-2. As these effects are mimicked by genetically or pharmacologically silencing Bcl-2, we conclude that the HDAC inhibitor-mediated induction of several apoptotic proteins and reversal of Bcl-2 upregulation through ER ensues cell death in our Tam-resistant cells. Though apparently high, the dose of 10 μM Tam used in our studies is justified based on the fact that the experimental media contains phenol red with pro-estrogenic properties, hence reducing Tam’s effective dose. Moreover, clinical trials in patients with high-dose Tam have reported approximately 3 to 4 μM plasma levels of Tam achieved without dose-limiting toxicity [32,33]. We recognize the limitation of not using a parallel passaged MCF7 control for this study.

In TAMR^M^ cells, we demonstrate that ERE-mediated transactivation is altered and that the ER has become less sensitive to ligands compared to MCF7 cells. The classic ER response genes PGR and TFF1 are substantially reduced in our model, consistent with previous findings [16,17,34]. As with the MCF7-derived TAMR^M^ cells, the T47D-derived TAMR^T^ cells also maintain ER expression, while losing PGR expression. The maintenance of ER expression following acquired resistance to anti-estrogens is consistent with breast tumors, as most retain ER expression (approximately 70 to 75%) [21,22,35]. The suppression of classic ER-response genes, PGR and TFF1, and lower basal luciferase activity at externally transfected ERE sites suggested reduced ER activity in TAMR^M^ cells despite elevated ER expression. Nonetheless, ER-mediated genomic signaling was not suppressed, but redirected, compared to MCF7 cells. A similar alteration of the ER transcriptome through a post translationally modified ER has been demonstrated recently by de Leeuw *et al.* [36]. Interestingly, the ER retained its transactivation function at most target genes that we evaluated. Furthermore, both TAMR^M^ and TAMR^T^ cells exhibited elevated BCL-2 mRNA and protein. However, despite the c-MYC oncogene being elevated in TAMR^M^ cells, ER did not regulate its transcription in contrast to MCF7 cells [37]. Our results show that ER is necessary for the proliferation of resistant cells signifying the retained importance of the ER in driving growth of these cells. Since recent studies suggest that mutations in the ligand-binding domain of ER may play an important role in the emergence of hormonal therapy resistance, we tested the possibility of point mutations in the ER in our resistant cells, by sequencing the entire gene, but found none [38].

ER-HDAC crosstalk and modulation of both ER expression and stability by HDAC inhibitors has been reported [26,39]. We demonstrate that the HDAC inhibitor, PCI, efficiently reduces ER mRNA and protein in ER-positive TAMR^M^ cells. We speculate this reduction is through similar mechanisms reported by other groups including pan-HDAC inhibitor-mediated induction of transcriptional repressor proteins, local methylation of CpG islands, influence on ER mRNA stability or through Hsp90 hyperacetylation resulting in loss of ER protein stability [40-42]. To the contrary, HDAC inhibition in ER-negative breast cancer cells, however, induces ER expression either through reversal of promoter hypermethylation or de-repression of ER mRNA via hyperactivated MAPK [43,44]. Previous studies have shown that HDAC inhibition induces expression of CDK inhibitors, such as p21, which causes cell cycle arrest in breast carcinomas [45,46]. PCI treatment similarly increases the CDK inhibitor p21 and causes G1 arrest in our resistant cells. Furthermore, HDAC inhibition also downregulates the elevated c-Myc in the TAMR^M^ cells. Thus, HDAC inhibitor-mediated epigenetic modulation reduces proliferation in TAMR^M^ cells.

Tumors employ diverse means to evade apoptosis, including modulation of Bcl-2 [47]. ER induces transcription of Bcl-2 in breast cancer cells upon estrogen stimulation [23]. In several clinical studies, increased Bcl-2 expression in breast tumors has correlated with favorable response to endocrine therapy [48,49]. Johnston *et al.* have reported that Tam treatment resulted in increased Bcl-2 expression in breast tumors, which, however, correlated with reduced Ki67 index or decreased cell proliferation [50]. Planas-Silva *et al.* analyzed tumor samples following progression on adjuvant hormonal therapy and reported increased Bcl-2 and c-Myc expression in metastatic lesions, compared to the primary tumor. In contrast to this finding, Gutierrez *et al.* analyzed tumors biopsied from local relapses while still on hormonal therapy and reported no increase in Bcl-2 expression upon tamoxifen treatment failure. Furthermore, the positive correlation between ER and Bcl-2 in primary tumors was lost following progression on hormonal therapy [51,52]. These clinical studies illustrate the complexity regarding Bcl-2 expression and hormonal therapy resistance. Although both directly compared Bcl-2 levels in the primary tumor and in tumors following hormonal therapy failure, they differed in terms of the site of recurrence, local versus distal metastasis. Furthermore, in one study, tumors were collected post endocrine treatment, whereas the other study was during continuation of Tam therapy, leading to the possibility of ER-dependent or ER-independent modulation of Bcl-2. Our Tam-resistant models exhibit Bcl-2 upregulation and maintenance of a positive correlation between ER and Bcl-2 post resistance, similar to the Planas-Silva study, suggesting Bcl-2 upregulation is an important phenomenon in metastatic Tam-resistant tumors. Further, we demonstrate the causal role for Bcl-2 upregulation in hormone therapy resistance, as its direct inhibition by an HDAC inhibitor PCI and Tam via the ER induces apoptosis, emphasizing the relevance of inhibiting both Bcl-2 and HDACs. Although very high levels of E2 can stimulate BCL-2 transactivation through the ER, premenopausal levels of E2 have a negligible effect on BCL-2 expression. This is in contrast to the significant increase in BCL-2 expression elicited by E2 in MCF7 cells. This further demonstrates the reduced sensitivity of ER to ligands in the Tam-resistant cells.

We have previously reported that co-administration of the HDAC inhibitor valproic acid and Tam, in Tam-sensitive T47D cells, leads to depletion of Bcl-2 protein [27]. From results in the current study, we conclude that the reduction of Bcl-2 mRNA by HDAC inhibition alone is modest, but when combined with Tam, its expression is significantly downregulated. We show that HDAC inhibition results in ER loss leading to decreased Bcl-2 expression. We speculate that tamoxifen further blocks residual ER activity at Bcl-2 promoter strongly eliminating Bcl-2 protein. However, studies detailing the underlying mechanism of BCL-2 downregulation using this therapeutic combination are ongoing.

The ability of PCI to induce cell death in Tam-resistant cells when Bcl-2 activity is repressed either genetically or pharmacologically emphasizes the importance of Bcl-2 as a target in Tam resistance. Recent studies of Bcl-2-specific inhibitors, in both *in vitro* models as well as in primary breast tumor xenografts that overexpress Bcl-2, have shown they potentiate apoptosis when combined with Tam [31]. Our data provides compelling evidence that HDAC inhibitors should be used in combination with Tam or a Bcl-2-specific inhibitor against tumors with elevated Bcl-2. Since ER drives resistance through modulation of Bcl-2, continued suppression of ER-mediated transactivation is likely important. The amount of cell death induced in MCF7 cells by co-administration of ABT.263 and PCI is similar to that in TAMR^M^ cells (Figure 7B), raising the possibility that targeting Bcl-2 and HDACs may also be an effective approach against tumors that do not exhibit elevated Bcl-2. As Bcl-2 is a key gatekeeper, countering apoptotic induction, it is not surprising that inhibiting it (for example pharmacologically or genetically) in the Tam-sensitive and -resistant cells tips the balance of both toward apoptosis when combined with the pro-apoptotic effects of HDAC inhibition. However, in this context, the effectiveness in both MCF7 and TAMR^M^ cells may be attributed to ABT.263’s ability to target Bcl-2 and its family members, Bcl-xl and Bcl-w, and by inhibiting the complete family, apoptosis may be induced irrespective of differing Bcl-2 levels. In support of this explanation, siRNA-mediated depletion of Bcl-2 enhanced apoptosis, when combined with PCI, only in the BCL-2-overexpressed TAMR^M^ cells (Figure 7C), but not in the MCF7 cells.

## Conclusions

The TAMR^M^ and TAMR^T^ cells represent a novel model of Tam-resistant ER-positive/PGR-negative breast cancer that exhibits elevated ER and Bcl-2 expression. This study emphasizes the importance of Bcl-2 elevation in acquired Tam resistance. It demonstrates that Bcl-2 downregulation and induction of pro-apoptotic proteins by combined ER and HDAC inhibition leads to apoptotic cell death of Tam-resistant cells. Inhibition of ER alone is sufficient to reduce growth, but not achieve cell death. Combined HDAC and ER inhibition inhibits proliferation and induces apoptosis by reversing changes to p21, c-Myc, and Bcl-2 expression and inducing pro-apoptotic factors. Thus, this work suggests that adding an HDAC inhibitor to anti-estrogen therapy may be effective in treating Tam-resistant ER-positive disease with elevated Bcl-2 expression.

## Additional files

Additional file 1: Figure S1. TAMR^M^ cells form tumors in mice in absence of an estradiol pellet. TAMR^M^ cells were grown in phenol red-free media supplemented with charcoal dextran stripped serum for 7 days before implanting subcutaneously in two mice for tumor formation. Tumor growth in absence of estradiol pellet was evaluated over time by caliper measurement and calculating volume as 0.5 (tumor length x tumor width^2^). Additional file 2: Figure S2. PCI-24781 induces cell death in TAMR^M^ cells genetically silenced for ER. TAMR^M^ cells were transfected with ESR1-directed siRNA and subsequently treated with PCI-24781 for 72 hours. Cells were then evaluated for viability **(A)** and PARP cleavage **(B)**. Additional file 3: Figure S3. The HDAC inhibitor PCI-24781 negatively regulates ER mRNA. TAMR^M^ cells were treated with either **(A)** increasing concentrations of the HDAC inhibitor PCI-24781 for 24 hours or **(B)** with 200 nM of PCI-24781 for 0, 2, 4, 10 and 24 hours, after which ER mRNA expression was evaluated. Additional file 4: Figure S4. PCI-24781 reduces BCL-2 mRNA in a dose-dependent manner. TAMR^M^ cells were treated with increasing concentrations of PCI-24781 (0, 200, 400, 800 nM) for 24 hours and BCL-2 mRNA expression was evaluated. Additional file 5: Figure S5. TAMR^M^ cells exhibit decreased sensitivity to apoptotic stress compared to MCF7 cells. MCF7 and TAMR^M^ cells were treated with increasing concentrations of **(A)** Tam and **(B)** doxorubicin for 72 hours and evaluated for cell death by trypan blue dye exclusion assay.

## Abbreviations

DMEM: Dulbecco’s modified Eagle’s medium
E2: estradiol
ER: estrogen receptor
ERE: estrogen response element
Ful: fulvestrant
HDAC: histone deacetylase
PBS: phosphate-buffered saline
PCI: PCI-24781
PGR: progesterone receptor
PT: PCI-24781 and 4-hydroxy tamoxifen
siRNA: small interfering RNA
Tam: 4-hydroxy tamoxifen
TAMR^M^: tamoxifen-resistant cells derived from MCF7
TAMR^T^: tamoxifen-resistant cells derived from T47D

## References

[1] Raha P, Thomas S, Munster PN (2011). Epigenetic modulation: a novel therapeutic target for overcoming hormonal therapy resistance. Epigenomics..

[2] Osborne CK, Schiff R (2011). Mechanisms of endocrine resistance in breast cancer. Annu Rev Med..

[3] Miller TW, Balko JM, Ghazoui Z, Dunbier A, Anderson H, Dowsett M (2011). A gene expression signature from human breast cancer cells with acquired hormone independence identifies MYC as a mediator of antiestrogen resistance. Clin Cancer Res..

[4] Pathiraja TN, Stearns V, Oesterreich S (2010). Epigenetic regulation in estrogen receptor positive breast cancer–role in treatment response. J Mammary Gland Biol Neoplasia..

[5] Kurebayashi J (2003). Endocrine-resistant breast cancer: underlying mechanisms and strategies for overcoming resistance. Breast Cancer..

[6] West AC, Johnstone RW (2014). New and emerging HDAC inhibitors for cancer treatment. J Clin Invest..

[7] Thurn KT, Thomas S, Moore A, Munster PN (2011). Rational therapeutic combinations with histone deacetylase inhibitors for the treatment of cancer. Future Oncol..

[8] Hirokawa Y, Arnold M, Nakajima H, Zalcberg J, Maruta H (2005). Signal therapy of breast cancers by the HDAC inhibitor FK228 that blocks the activation of PAK1 and abrogates the tamoxifen-resistance. Cancer Biol Ther..

[9] Jang ER, Lim SJ, Lee ES, Jeong G, Kim TY, Bang YJ (2004). The histone deacetylase inhibitor trichostatin A sensitizes estrogen receptor alpha-negative breast cancer cells to tamoxifen. Oncogene..

[10] Matthews GM, Newbold A, Johnstone RW (2012). Intrinsic and extrinsic apoptotic pathway signaling as determinants of histone deacetylase inhibitor antitumor activity. Adv Cancer Res..

[11] Munster PN, Thurn KT, Thomas S, Raha P, Lacevic M, Miller A (2011). A phase II study of the histone deacetylase inhibitor vorinostat combined with tamoxifen for the treatment of patients with hormone therapy-resistant breast cancer. Br J Cancer..

[12] Yardley DA, Ismail-Khan RR, Melichar B, Lichinitser M, Munster PN, Klein PM (2013). Randomized phase II, double-blind, placebo-controlled study of exemestane with or without entinostat in postmenopausal women with locally recurrent or metastatic estrogen receptor-positive breast cancer progressing on treatment with a nonsteroidal aromatase inhibitor. J Clin Oncol..

[13] Thomas S, Thurn KT, Raha P, Chen S, Munster PN (2013). Efficacy of histone deacetylase and estrogen receptor inhibition in breast cancer cells due to concerted down regulation of Akt. PLoS One..

[14] Nawata H, Bronzert D, Lippman ME (1981). Isolation and characterization of a tamoxifen-resistant cell line derived from MCF-7 human breast cancer cells. J Biol Chem..

[15] Nawata H, Chong MT, Bronzert D, Lippman ME (1981). Estradiol-independent growth of a subline of MCF-7 human breast cancer cells in culture. J Biol Chem..

[16] Massarweh S, Osborne CK, Creighton CJ, Qin L, Tsimelzon A, Huang S (2008). Tamoxifen resistance in breast tumors is driven by growth factor receptor signaling with repression of classic estrogen receptor genomic function. Cancer Res..

[17] Stone A, Valdes-Mora F, Gee JM, Farrow L, McClelland RA, Fiegl H (2012). Tamoxifen-induced epigenetic silencing of oestrogen-regulated genes in anti-hormone resistant breast cancer. PLoS One..

[18] Thrane S, Lykkesfeldt AE, Larsen MS, Sorensen BS, Yde CW (2013). Estrogen receptor alpha is the major driving factor for growth in tamoxifen-resistant breast cancer and supported by HER/ERK signaling. Breast Cancer Res Treat..

[19] Adamo V, Iorfida M, Montalto E, Festa V, Garipoli C, Scimone A, et al. Overview and new strategies in metastatic breast cancer (MBC) for treatment of tamoxifen-resistant patients. Ann Oncol. 2007; 18:vi53–7. 10.1093/annonc/mdm225 17591833

[20] Osborne CK, Wakeling A, Nicholson RI (2004). Fulvestrant: an oestrogen receptor antagonist with a novel mechanism of action. Br J Cancer..

[21] Osborne CK (1998). Tamoxifen in the treatment of breast cancer. N Engl J Med..

[22] Johnston SR, Saccani-Jotti G, Smith IE, Salter J, Newby J, Coppen M (1995). Changes in estrogen receptor, progesterone receptor, and pS2 expression in tamoxifen-resistant human breast cancer. Cancer Res..

[23] Dong L, Wang W, Wang F, Stoner M, Reed JC, Harigai M (1999). Mechanisms of transcriptional activation of bcl-2 gene expression by 17beta-estradiol in breast cancer cells. J Biol Chem..

[24] Dubik D, Shiu RP (1988). Transcriptional regulation of c-myc oncogene expression by estrogen in hormone-responsive human breast cancer cells. J Biol Chem..

[25] Mandal S, Davie JR (2010). Estrogen regulated expression of the p21 Waf1/Cip1 gene in estrogen receptor positive human breast cancer cells. J Cell Physiol..

[26] Bicaku E, Marchion DC, Schmitt M, Munster PN (2008). Selective inhibition of histone deacetylase 2 silences progesterone receptor mediated signaling. Cancer Res..

[27] Thomas S, Thurn KT, Bicaku E, Marchion DC, Munster PN (2011). Addition of a histone deacetylase inhibitor redirects tamoxifen-treated breast cancer cells into apoptosis, which is opposed by the induction of autophagy. Breast Cancer Res Treat..

[28] Kim YH, Park JW, Lee JY, Surh YJ, Kwon TK (2003). Bcl-2 overexpression prevents daunorubicin-induced apoptosis through inhibition of XIAP and Akt degradation. Biochem Pharmacol..

[29] Raisova M, Goltz G, Bektas M, Bielawska A, Riebeling C, Hossini AM (2002). Bcl-2 overexpression prevents apoptosis induced by ceramidase inhibitors in malignant melanoma and HaCaT keratinocytes. FEBS Lett..

[30] Wang Y, Wang X, Zhao H, Liang B, Du Q (2012). Clusterin confers resistance to TNF-alpha-induced apoptosis in breast cancer cells through NF-kappaB activation and Bcl-2 overexpression. J Chemother..

[31] Vaillant F, Merino D, Lee L, Breslin K, Pal B, Ritchie ME (2013). Targeting BCL-2 with the BH3 mimetic ABT-199 in estrogen receptor-positive breast cancer. Cancer Cell..

[32] Trump DL, Smith DC, Ellis PG, Rogers MP, Schold SC, Winer EP (1992). High-dose oral tamoxifen, a potential multidrug-resistance-reversal agent: phase I trial in combination with vinblastine. J Natl Cancer Inst..

[33] Bergan RC, Reed E, Myers CE, Headlee D, Brawley O, Cho HK (1999). A Phase II study of high-dose tamoxifen in patients with hormone-refractory prostate cancer. Clin Cancer Res..

[34] Badia E, Duchesne MJ, Fournier-Bidoz S, Simar-Blanchet AE, Terouanne B, Nicolas JC (1994). Hydroxytamoxifen induces a rapid and irreversible inactivation of an estrogenic response in an MCF-7-derived cell line. Cancer Res..

[35] Brunner N, Frandsen TL, Holst-Hansen C, Bei M, Thompson EW, Wakeling AE (1993). MCF7/LCC2: a 4-hydroxytamoxifen resistant human breast cancer variant that retains sensitivity to the steroidal antiestrogen ICI 182,780. Cancer Res..

[36] de Leeuw R, Flach K, Bentin Toaldo C, Alexi X, Canisius S, Neefjes J (2013). PKA phosphorylation redirects ERalpha to promoters of a unique gene set to induce tamoxifen resistance. Oncogene..

[37] Wang C, Mayer JA, Mazumdar A, Fertuck K, Kim H, Brown M (2011). Estrogen induces c-myc gene expression via an upstream enhancer activated by the estrogen receptor and the AP-1 transcription factor. Mol Endocrinol..

[38] Toy W, Shen Y, Won H, Green B, Sakr RA, Will M (2013). ESR1 ligand-binding domain mutations in hormone-resistant breast cancer. Nat Genet..

[39] deFazio A, Chiew YE, Donoghue C, Lee CS, Sutherland RL. Effect of sodium butyrate on estrogen receptor and epidermal growth factor receptor gene expression in human breast cancer cell lines. J Biol Chem. 1992; 267:18008–12. 1517234

[40] Fiskus W, Ren Y, Mohapatra A, Bali P, Mandawat A, Rao R (2007). Hydroxamic acid analogue histone deacetylase inhibitors attenuate estrogen receptor-alpha levels and transcriptional activity: a result of hyperacetylation and inhibition of chaperone function of heat shock protein 90. Clin Cancer Res..

[41] Oie S, Matsuzaki K, Yokoyama W, Murayama A, Yanagisawa J (2013). HDAC3 regulates stability of estrogen receptor alpha mRNA. Biochem Biophys Res Commun..

[42] Reid G, Metivier R, Lin CY, Denger S, Ibberson D, Ivacevic T (2005). Multiple mechanisms induce transcriptional silencing of a subset of genes, including oestrogen receptor alpha, in response to deacetylase inhibition by valproic acid and trichostatin A. Oncogene..

[43] Sharma D, Blum J, Yang X, Beaulieu N, Macleod AR, Davidson NE (2005). Release of methyl CpG binding proteins and histone deacetylase 1 from the Estrogen receptor alpha (ER) promoter upon reactivation in ER-negative human breast cancer cells. Mol Endocrinol..

[44] Plotkin A, Volmar CH, Wahlestedt C, Ayad N, El-Ashry D (2014). Transcriptional repression of ER through hMAPK dependent histone deacetylation by class I HDACs. Breast Cancer Res Treat..

[45] Ocker M, Schneider-Stock R (2007). Histone deacetylase inhibitors: signalling towards p21cip1/waf1. Int J Biochem Cell Biol..

[46] Duong V, Bret C, Altucci L, Mai A, Duraffourd C, Loubersac J (2008). Specific activity of class II histone deacetylases in human breast cancer cells. Mol Cancer Res..

[47] Strasser A, Cory S, Adams JM (2011). Deciphering the rules of programmed cell death to improve therapy of cancer and other diseases. EMBO J..

[48] Silvestrini R, Benini E, Veneroni S, Daidone MG, Tomasic G, Squicciarini P (1996). p53 and bcl-2 expression correlates with clinical outcome in a series of node-positive breast cancer patients. J Clin Oncol..

[49] Hellemans P, van Dam PA, Weyler J, van Oosterom AT, Buytaert P, Van Marck E (1995). Prognostic value of bcl-2 expression in invasive breast cancer. Br J Cancer..

[50] Johnston SR, MacLennan KA, Sacks NP, Salter J, Smith IE, Dowsett M (1994). Modulation of Bcl-2 and Ki-67 expression in oestrogen receptor-positive human breast cancer by tamoxifen. Eur J Cancer..

[51] Planas-Silva MD, Bruggeman RD, Grenko RT, Smith JS (2007). Overexpression of c-Myc and Bcl-2 during progression and distant metastasis of hormone-treated breast cancer. Exp Mol Pathol..

[52] Gutierrez MC, Detre S, Johnston S, Mohsin SK, Shou J, Allred DC (2005). Molecular changes in tamoxifen-resistant breast cancer: relationship between estrogen receptor, HER-2, and p38 mitogen-activated protein kinase. J Clin Oncol..

