# Supplementary material for: Combined histone deacetylase inhibition and tamoxifen induces apoptosis in tamoxifen-resistant breast cancer models, by reversing Bcl-2 overexpression
Source: Breast Cancer Res. 2015 Feb 25;17(1):26. doi: 10.1186/s13058-015-0533-z (PMC4367983; doi:10.1186/s13058-015-0533-z)

Figure S1: TAMR^{M} cells form tumors in mice in absence of an estradiol pellet.

TAMR^{M} cells were grown in phenol-red free media supplemented with charcoal dextran stripped serum for 7 days before implanting subcutaneously in two mice for tumor formation. Tumor growth in absence of estradiol pellet was evaluated over time by caliper measurement and calculating volume as 0.5 (tumor length x tumor width^{2}).

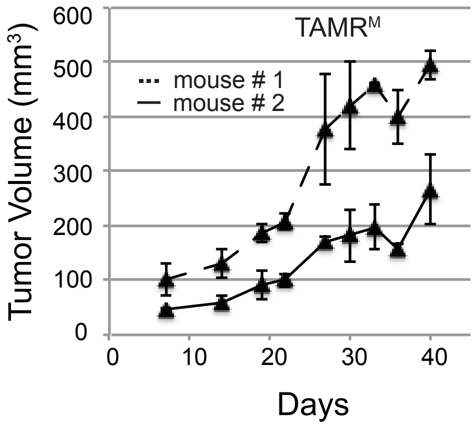

Supplement: Additional file 1: Figure S1. — TAMRM cells form tumors in mice in absence of an estradiol pellet. TAMRM cells were grown in phenol red-free media supplemented with charcoal dextran stripped serum for 7 days before implanting subcutaneously in two mice for tumor formation. Tumor growth in absence of estradiol pellet was evaluated over time by caliper measurement and calculating volume as 0.5 (tumor length x tumor width2). [file 13058_2015_533_MOESM1_ESM.pdf]
