# Supplementary material for: Combined histone deacetylase inhibition and tamoxifen induces apoptosis in tamoxifen-resistant breast cancer models, by reversing Bcl-2 overexpression
Source: Breast Cancer Res. 2015 Feb 25;17(1):26. doi: 10.1186/s13058-015-0533-z (PMC4367983; doi:10.1186/s13058-015-0533-z)

Figure S2: PCI-24781 induces cell death in TAMR^{M} cells genetically silenced for ER.

TAMR^{M} cells were transfected with ESR1 directed siRNA and subsequently treated with PCI-24781 for 72 hours. Cells were then evaluated for viability (A) and PARP cleavage (B).

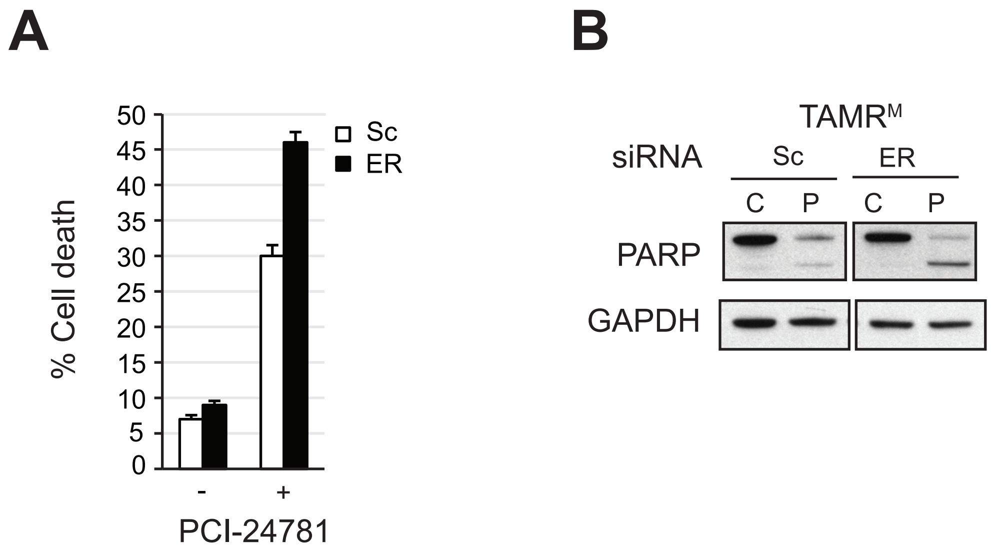

Supplement: Additional file 2: Figure S2. — PCI-24781 induces cell death in TAMRM cells genetically silenced for ER. TAMRM cells were transfected with ESR1-directed siRNA and subsequently treated with PCI-24781 for 72 hours. Cells were then evaluated for viability (A) and PARP cleavage (B). [file 13058_2015_533_MOESM2_ESM.pdf]
