# Supplementary material for: Combined histone deacetylase inhibition and tamoxifen induces apoptosis in tamoxifen-resistant breast cancer models, by reversing Bcl-2 overexpression
Source: Breast Cancer Res. 2015 Feb 25;17(1):26. doi: 10.1186/s13058-015-0533-z (PMC4367983; doi:10.1186/s13058-015-0533-z)

Figure S3: The HDAC inhibitor PCI-24781 negatively regulates ER mRNA.

TAMR[M] cells were treated with either (A) increasing concentrations of the HDAC inhibitor PCI-24781 for 24 hours or (B) with 200 nM of PCI-24781 for 0, 2, 4, 10 and 24 hours, after which ER mRNA expression was evaluated.

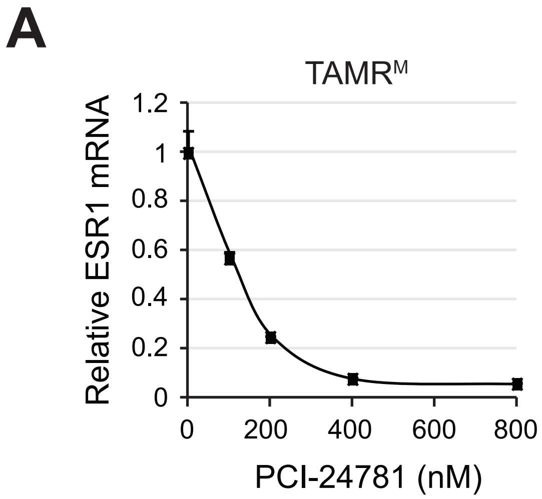

Supplement: Additional file 3: Figure S3. — The HDAC inhibitor PCI-24781 negatively regulates ER mRNA. TAMRM cells were treated with either (A) increasing concentrations of the HDAC inhibitor PCI-24781 for 24 hours or (B) with 200 nM of PCI-24781 for 0, 2, 4, 10 and 24 hours, after which ER mRNA expression was evaluated. [file 13058_2015_533_MOESM3_ESM.pdf]
