# Supplementary material for: Combined histone deacetylase inhibition and tamoxifen induces apoptosis in tamoxifen-resistant breast cancer models, by reversing Bcl-2 overexpression
Source: Breast Cancer Res. 2015 Feb 25;17(1):26. doi: 10.1186/s13058-015-0533-z (PMC4367983; doi:10.1186/s13058-015-0533-z)

Figure S4: PCI-24781 reduces BCL-2 mRNA in a dose dependent manner.

TAMR^{M} cells were treated with increasing concentrations of PCI-24781 (0, 200, 400, 800 nM) for 24 hours and BCL-2 mRNA expression was evaluated.

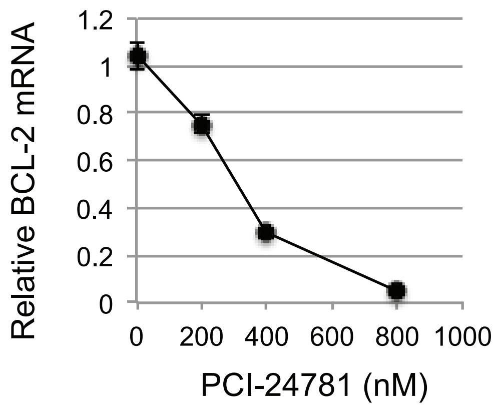

Supplement: Additional file 4: Figure S4. — PCI-24781 reduces BCL-2 mRNA in a dose-dependent manner. TAMRM cells were treated with increasing concentrations of PCI-24781 (0, 200, 400, 800 nM) for 24 hours and BCL-2 mRNA expression was evaluated. [file 13058_2015_533_MOESM4_ESM.pdf]
