# Supplementary material for: Combined histone deacetylase inhibition and tamoxifen induces apoptosis in tamoxifen-resistant breast cancer models, by reversing Bcl-2 overexpression
Source: Breast Cancer Res. 2015 Feb 25;17(1):26. doi: 10.1186/s13058-015-0533-z (PMC4367983; doi:10.1186/s13058-015-0533-z)

Figure S5: TAMR[M] cells exhibit decreased sensitivity to apoptotic stress as compared to MCF7.

MCF7 and TAMR[M] cells were treated with increasing concentrations of (A) Tam and (B) doxorubicin for 72 hours and evaluated for cell death by trypan blue dye exclusion assay.

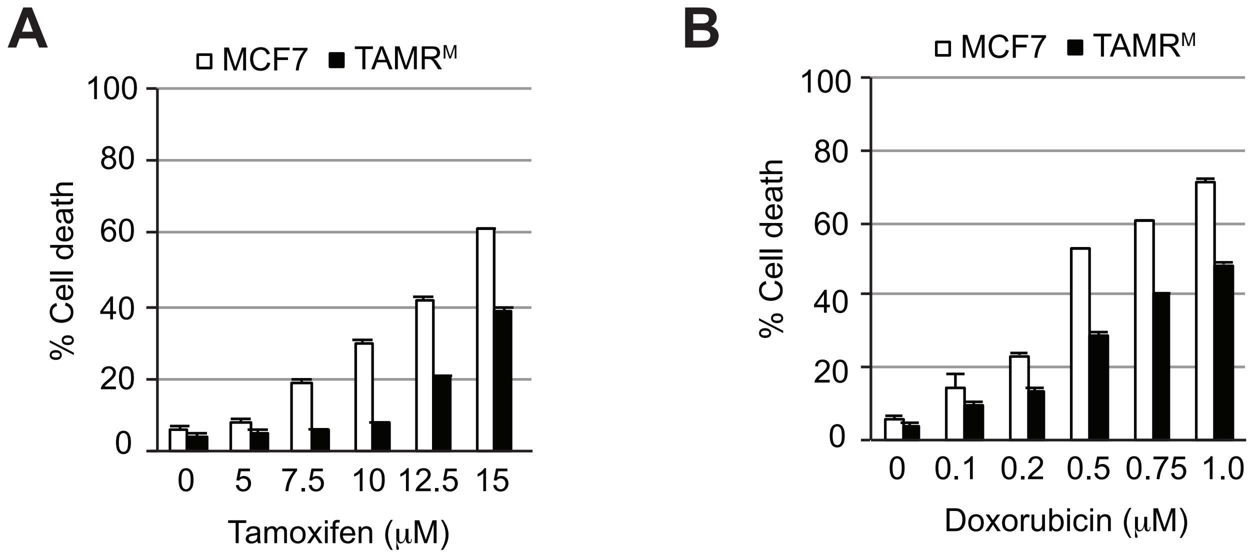

Supplement: Additional file 5: Figure S5. — TAMRM cells exhibit decreased sensitivity to apoptotic stress compared to MCF7 cells. MCF7 and TAMRM cells were treated with increasing concentrations of (A) Tam and (B) doxorubicin for 72 hours and evaluated for cell death by trypan blue dye exclusion assay. [file 13058_2015_533_MOESM5_ESM.pdf]
